# Identification of Monobenzone as a Novel Potential Anti-Acute Myeloid Leukaemia Agent That Inhibits RNR and Suppresses Tumour Growth in Mouse Xenograft Model

**DOI:** 10.3390/cancers14194710

**Published:** 2022-09-27

**Authors:** Jingwen Dong, Tingting Zhong, Zhijian Xu, Haiyi Chen, Xianjun Wang, Lili Yang, Zhiyuan Lou, Yuanling Xu, Tingjun Hou, Rongzhen Xu, Weiliang Zhu, Jimin Shao

**Affiliations:** 1 Department of Pathology and Pathophysiology, Cancer Institute of the Second Affiliated Hospital, School of Medicine, Zhejiang University, Hangzhou 310005, China; 2 Key Laboratory of Disease Proteomics of Zhejiang Province, Key Laboratory of Cancer Prevention and Intervention of China National Ministry of Education, School of Medicine, Zhejiang University, Hangzhou 310030, China; 3 Cancer Center, Zhejiang University, Hangzhou 310030, China; 4 Department of Pathology of Sir Run Run Shaw Hospital, School of Medicine, Zhejiang University, Hangzhou 310020, China; 5 Drug Discovery and Design Center, Shanghai Institute of Materia Medica, Chinese Academy of Sciences, Shanghai 310063, China; 6 Hangzhou Institute of Innovative Medicine, College of Pharmaceutical Sciences, Zhejiang University, Hangzhou 310030, China

**Keywords:** acute myeloid leukaemia, ribonucleotide reductase, monobenzone, anti-proliferative activity, combination therapy

## Abstract

**Simple Summary:**

The clinical treatment of acute myeloid leukaemia is still dominated by chemotherapy. Clinically used anti-leukaemia drugs have shortcomings such as myelosuppression, toxicity and drug resistance. Therefore, the need to develop other chemotherapeutic drugs to meet more clinical needs is urgent. Ribonucleotide reductase (RNR) consists of a catalytic large subunit M1 (RRM1) and a regulatory small subunit M2 (RRM2), which provides dNTPs for DNA synthesis. The rapid proliferation of cancer cells requires large amounts of dNTPs. Therefore, the use of RNR inhibitors is a promising strategy for the clinical treatment of various malignancies. Monobenzone is an FDA-approved depigmenting agent for vitiligo patients. In this study, we demonstrate that monobenzone is a potent inhibitor of RNR enzyme activity by targeting RRM2 protein, and thus has significant anti-leukaemia efficacy in vitro and in vivo. This finding suggests that monobenzone has the potential to be optimized as a novel anti-AML therapeutic drug in the future.

**Abstract:**

Acute myeloid leukaemia (AML) is one of the most common types of haematopoietic malignancy. Ribonucleotide reductase (RNR) is a key enzyme required for DNA synthesis and cell proliferation, and its small subunit RRM2 plays a key role for the enzymatic activity. We predicted monobenzone (MB) as a potential RRM2 target compound based on the crystal structure of RRM2. In vitro, MB inhibited recombinant RNR activity (IC50 = 0.25 μM). Microscale thermophoresis indicated that MB inhibited RNR activity by binding to RRM2. MB inhibited cell proliferation (MTT IC50 = 6–18 μM) and caused dose-dependent DNA synthesis inhibition, cell cycle arrest, and apoptosis in AML cells. The cell cycle arrest was reversed by the addition of deoxyribonucleoside triphosphates precursors, suggesting that RNR was the intracellular target of the compound. Moreover, MB overcame drug resistance to the common AML drugs cytarabine and doxorubicin, and treatment with the combination of MB and the Bcl-2 inhibitor ABT-737 exerted a synergistic inhibitory effect. Finally, the nude mice xenografts study indicated that MB administration produced a significant inhibitory effect on AML growth with relatively weak toxicity. Thus, we propose that MB has the potential as a novel anti-AML therapeutic agent in the future.

## 1. Introduction

Acute myeloid leukaemia (AML) is a blood cancer characterized by abnormal proliferation and differentiation arrest of myeloid progenitor cells, and it is one of the most common types of haematopoietic malignancy. Due to the rapid onset of the pathogenesis of several subtypes of AML and the lack of specific therapies, patients must undergo treatment for a long time, and more than half of patients ultimately die from their disease [1,2,3]. The current clinical treatment strategies for the disease include chemotherapy, radiotherapy, immunotherapy, and bone marrow transplantation. Among them, the most common treatment used over the last decades is “7 + 3” standard induction chemotherapy, consisting of cytarabine and daunorubicin. However, these anti-leukaemia drugs have limitations, such as low specificity, myelosuppression, hepatotoxicity, nephrotoxicity, and gastrointestinal toxicity, which may cause intolerance in patients [4,5,6,7,8]. Meanwhile, leukaemia cells tend to exhibit chemoresistance after a period of treatment due to the abnormal expression of drug resistance-related proteins, the dysregulation of certain enzyme activities, mutations in oncogenes and chemo-induced DNA damage repair [9,10,11,12,13]. As a result, patients with AML usually experience high risks of relapse or developing secondary tumours. Therefore, developing novel anti-leukaemia agents with high potency and few side effects is still challenging.

Ribonucleotide reductase (RNR) catalyses the conversion of ribonucleoside diphosphates (NDPs) into deoxyribonucleoside diphosphates (dNDPs), which are then phosphorylated to become deoxyribonucleoside triphosphates (dNTPs), the building blocks for DNA synthesis in cells. The RNR holoenzyme consists of the catalytic large subunit M1 (RRM1) and the regulatory small subunit M2 (RRM2) or its homologue RRM2B [14,15,16]. The fast proliferation of cancer cells requires a large number of dNTPs for DNA replication and repair. Pan-cancer expression profiling studies have revealed that the expression of RRM2 and RRM1 is upregulated in multiple types of cancers [17,18,19]. The use of RNR inhibitors, either as a single agent or combined with other therapies, has been suggested to be a promising strategy for the clinical treatment of multiple malignancies [20,21,22]. Several RNR inhibitors are also used in anti-leukaemia therapies, such as hydroxyurea (HU) that targets RRM2 to treat chronic myeloid leukaemia (CML) and AML and the ribonucleoside analogues cytarabine and clofarabine that target RRM1 to treat acute leukaemia [23,24,25]. However, these RNR inhibitors still limit their clinical application [22,26,27]. Thus, novel categories of RNR inhibitors may meet this urgent clinical need.

Monobenzone (monobenzyl ether of hydroquinone, MB) is a depigmenting agent that was discovered by Oliver et al. in 1939 [28]. It has been approved by the FDA as a cream formulation for skin depigmentation of patients with vitiligo. The mechanism of action for depigmentation by MB is correlated with the inhibition of tyrosinase [29,30,31]. The compound also is cytotoxic to melanocytes and melanoma cells and increases melanocyte and melanoma cell immunogenicity [32,33,34,35,36]. In this study, we show that RRM2 expression is strongly correlated with malignant proliferation in patients with AML and that MB is a potent inhibitor of RNR enzymatic activity by targeting the RRM2 protein, thereby possessing significant anti-leukaemia efficacy in vitro and in vivo. The compound may overcome the resistance to cytarabine (Ara-C) and doxorubicin (DOX), and its combined use with pro-apoptotic agents significantly enhances inhibitory effects on AML cells. This finding suggests the potential for MB to be optimized as a novel anti-AML therapeutic agent in the future.

## 2. Materials and Methods

### 2.1. Data Acquisition and Gene Set Enrichment Analysis (GSEA)

All expression datasets for patients with AML were downloaded from the GEO, TCGA and GTEx databases. The GSE147515 dataset was obtained from GEO, including 198 normal samples and 1534 samples from patients with AML retrieved from 11 datasets [37]. We also downloaded mRNA expression data and clinical information from 151 samples from patients with AML from TCGA (https://portal.gdc.cancer.gov, accessed on 21 February 2022).

GSEA was performed to interpret biological pathways related to RRM2 expression [38]. We divided the AML samples into two groups according to RRM2 expression levels (high- and low-RRM2 groups) and downloaded the c2.cp.kegg.v7.4.symbols.gmt subsets from the Molecular Signatures Database (MSigDB) to evaluate relevant pathways and molecular mechanisms. Gene sets with a normalized enrichment score |NES| > 1.0, NOM *p*-value < 0.05 and FDR *q*-value < 0.25 were considered statistically significantly enriched.

### 2.2. Similarity Search and Molecular Docking

Similarity searching of the approved drug from DrugBank release version 5.1.5 [39] against osalmid was performed by Open Babel 2.4.0 [40] using FP3 molecular fingerprints.

The crystal structure of the RRM2 protein was retrieved from the RCSB database (PDB ID: 3OLJ) [41]. The Protein Preparation Wizard in Schrödinger 2020 (www.schrodinger.com, accessed date: 21 January 2022) was used to remove the water molecules and ions, add hydrogens and fix bond orders in the crystal structure. The structure of MB was processed using the LigPrep module in Schrödinger 2020 (www.schrodinger.com, accessed date: 21 January 2022). The docking software Glide [42] was used to generate the RRM2-MB complex. The binding box with a size of 20 × 20 × 20 Å centred on D271 was generated using the Receptor Grid Generation component of Glide, and the ligand MB was docked to the box using the extra precision (XP) scoring function of Glide.

### 2.3. Compounds, Antibodies and shRNAs

HU and Ara-C were purchased from Sigma-Aldrich (St. Louis, MO, USA). MB, DOX and ABT-737 were purchased from Selleck (Shanghai, China).

Antibodies against RRM1 (sc-11733), RRM2 (sc-398294) and RRM2B (sc-10840) were purchased from Santa Cruz Biotech (Dallas, TX, USA). Antibodies against tubulin (ER130905) and GAPDH (EM1101) were purchased from HuaBio (Hangzhou, China). Antibodies against r-H2Ax (ab81299) were purchased from Abcam (Cambridge, UK). Antibodies against H3 (EM30605) were purchased from HuaBio (Hangzhou, China).

Short hairpin RNAs targeting RRM2 (shRRM2) and the negative control (shNC) were purchased from Huabio (Shanghai, China) and used to establish stably transfected KG-1A cell lines by applying 2 μg/mL puromycin selection after transfection.

### 2.4. Preparation of the Recombinant RNR Protein and Activity Assays

The expression and purification of RRM1 and RRM2 proteins and the RNR enzymatic activity assays were performed as previously described [15]. Different doses of compounds were incubated with 1 μM RRM1 and 1 μM RRM2 proteins at 25 °C for 30 min and then assayed for the enzymatic activity inhibition rate using the solvent DMSO as a negative control. The RNR activity was calculated as follows: RNR activity = dCDP/(CDP + dCDP) × 100%.

### 2.5. Microscale Thermophoresis (MST) Assays

MST experiments were performed using a Monolith NT.115Pico instrument (NanoTemper Technologies, Munich, Germany), ordinary capillary and 20% LED power. The data were analysed at medium MST power. All small molecules were stored in pure DMSO at a concentration of 100 µM at −20 °C. For MB, a 2-fold serial dilution was performed in 16 tubes with 10 μL in each tube using assay buffer (50 mM Tris-HCl, 100 mM KCl, 5 mM DTT, pH 7.6, supplemented with 0.1% Pluronic F-127). The highest concentration of MB in the first tube was 5 μM in 5% DMSO. Then, 10 μL of lysine-labelled RRM2 (200 nM) were added to each tube. After mixing and incubating at room temperature for 30 min, the mixture was transferred to a capillary. MO Control software was used to obtain data in Binding Check, Binding Affinity or Expert mode. The MST measurement was performed at 25 °C for 20 seconds. The K_D_ value was calculated based on the concentration-dependent change in RRM2 normalized fluorescence (Fnorm) in the presence of the compound after 10 s on time of the MST. The K_D_ value obtained was the average of three independent measurements. The data were analysed using MO affinity analysis software (NanoTemper Technologies). 

### 2.6. Cell Lines and Drug Resistance Induction

KG-1A, MOLM13, NB4, U2932, OCI-LY3, Jeko-1, and Ramos cells were kind gifts from Professor Rongzhen Xu at the Second Affiliated Hospital of Zhejiang University. U2661B and NCI-H929 cells were purchased from the Cell Bank at the Chinese Academy of Sciences (Shanghai, China) and National Experimental Cell Resource Sharing Service Platform, respectively. B16 cells were a kind gift from Professor Jian Sun of Zhejiang University. NB4, OCI-LY3, and U2661B cells were cultured in IMDM. Other cells were all cultured in RPMI 1640 medium. The complete medium contained 10% foetal bovine serum, 100 units/ml penicillin, and 100 units/mL streptomycin. All cells were cultured at 37 °C in a humidified atmosphere with 5% CO_2_.

Ara-C-, DOX-, and HU-resistant KG-1A cell lines (KG-1A-ARAC, KG-1A-DOX, and KG-1A-HU) were constructed by stepwise incubations with the respective drugs. Briefly, KG-1A cells in logarithmic growth phase were cultured with different 50% inhibitory concentrations (IC_50_s) of Ara-C, DOX, and HU (0.15, 0.05, 100 μM, respectively, diluted with serum-free RPMI 1640, prepared immediately before use). After the cells were confluent and stably passaged 4 times, the drug treatment concentration was increased to 1.5 times the original concentration, which was repeated until drug resistance developed and remained stable in drug-free culture medium. Drug resistance was determined by performing MTT assays every 4 weeks. The whole process lasted 6 months.

### 2.7. MTT Cell Viability Assays and Analyses of the Effects of Drug Combinations 

One hundred microliters of cells (5000–30,000 cells) were seeded into each well of 96-well plates and incubated with 100 μL of different doses of compounds for 72 h. Then, 20 μL of a 5 mg/mL MTT solution were added. After 4 h of incubation at 37 °C, the supernatant was removed, and 200 µL of solution (10% *m*/*v* SDS, 5% *v*/*v* isobutanol, and 0.1% 10 M HCl) were added to the well. After an overnight incubation at 37 °C, the absorbance (OD) of each well was measured at 570 nm using a microplate reader. The cell viabilities and IC_50_s were calculated using GraphPad Prism v6 software (GraphPad Software, San Diego, CA, USA). The cell viability of the solvent control group (0.1% DMSO in complete growth medium) was set to 100%.

For analyses of the effects of drug combinations, MB was mixed with ABT-737 at ratios of 20:1 and 5:1 according to their IC_50_s for MOLM13 and KG-1A cells, respectively, and two-fold serial dilutions covering their EC_90_, EC_75_, EC_50_ and EC_25_ values were added to the cell culture media, with 3 replicate wells analysed for each concentration of each ratio. On Day 3, cell viability was determined by performing MTT assays. The effects of drug combinations were determined using the median effect methods reported by Chou and Talalay with the CalcuSyn program (Biosoft, Cambridge, UK) [43].

### 2.8. Flow Cytometry Measurements

For the cell cycle analysis, tumour cells were treated with different doses of compounds for 24 h in 6-well plates. The cells were fixed with 70% ethyl alcohol at 4 °C overnight. Then, the samples were washed with PBS and stained with PI in buffer (2% *v*/*v* PI and 0.2% *v*/*v* RNase A, MULTI SCIENCES) for 15 min at room temperature. A flow cytometer (FC500 MPL Beckman Coulter) was used to determine the cell cycle distribution. For the assessment of the ability of dNTPs precursors deoxyribonucleoside (dNs) to reverse the effects of drugs, tumour cells were treated with different doses of compounds and dNs at a 1:2 ratio for 24 h in 6-well plates. The detection method is the same as described above.

For cell apoptosis analyses, tumour cells were treated with different doses of compounds for 48 h. The cells were collected and washed with PBS. Then, the cells were stained with an Annexin V-FITC solution (1% *v*/*v* Annexin V-FITC + 99% binding buffer, Multi Sciences, Hangzhou, China) for 30 min at room temperature, followed by a propidium iodide solution (Multi Sciences, Hangzhou, China) addition. Apoptosis was determined using a flow cytometer (FC500 MPL Beckman Coulter).

For EdU incorporation assays, cells were seeded and treated with several concentrations of compounds for 24 h in 6-well plates. Then, DNA synthesis was measured using the Click-iT Plus EdU Alexa Fluor 647 Flow Cytometry Assay Kit (Invitrogen, C10634, Waltham, MA, USA). The EdU incorporation ratio was detected using flow cytometry.

### 2.9. Quantitative Real-Time PCR and Western Blotting

Using RNAiso Plus (TaKaRa, Kusatsu, Shiga, Japan), PrimeScript RT kit (TaKaRa, Kusatsu, Shiga, Japan), SYBR^®^ Premix Ex TaqTM (TaKaRa, Kusatsu, Shiga, Japan) and LightCycler^®^ 480 system (Roche Diagnostics, Basel, Switzerland), the total RNA was extracted from cells and then reverse transcribed for quantitative real-time PCR (qRT-PCR). Gene expression was normalised using actin levels as an internal control.

For Western blots, cells were collected by centrifugation after washing twice with phosphate-buffered saline (PBS) and then lysed in RIPA lysis buffer (Millipore, Darmstadt, Germany) containing an intact protease inhibitor (Roche Basel, Switzerland) and a phosphatase inhibitor (Roche) to obtain whole cell extracts. Protein concentrations were measured using the Bradford method (Bio-Rad, Hercules, CA, USA). Extracts were separated on SDS-PAGE gels and transferred to nitrocellulose membranes (Whatman, Maidstone, UK), followed by incubation with diluted primary antibodies and then with IRDye 800CW or IRDye 680-conjugated secondary antibodies. The results were visualised using an Odyssey infrared imaging system (LI-COR Biosciences, Lincoln, NE, USA).

### 2.10. Tyrosinase Activity Assay

Tyrosinase activity was determined using the method described by Kim et al. [44], with slight modifications. Briefly, AML cells were plated in 96-well plates at a density of 15,000–20,000 cells/well. After an incubation with the test substance, the cells were washed with PBS, lysed with 100 µL of 1% Triton-X/PBS (*v*/*v*, 90 Al/well), and then frozen at −80 °C for 1–2 hours. The cells were thawed at room temperature for 40 minutes to rupture the cells. After thawing and mixing in a 37 °C water bath for 1 hour, 100 μL of 0.2% L-DOPA/PBS (*m*/*v*) were added to each well. After an incubation for 4–6 hours at 37 °C, the absorbance was measured at 475 nm.

### 2.11. Mouse Tumour Xenograft Experiments

Two hundred microlitres of PBS containing 2 × 10^5^ MOLM13 cells were injected subcutaneously into 4-week-old male nude mice (Shanghai SLAC Laboratory Animal Co., Ltd., Shanghai, China). After the xenografts were confirmed, the mice were randomly divided into three groups (N = 8 mice per group) and treated with the solvent (5% DMSO, 8% Tween 80, and 87% normal saline), 150 or 200 mg/kg MB, respectively, by daily intraperitoneal injection. The tumour size and the body weight of each mouse were measured daily. The tumour volume was calculated as 0.5ab2 (a = long diameter of the tumour, b = short diameter). After 2 weeks of treatment, the mice were sacrificed, and the tumour weights were measured. For each mouse, the alanine transaminase (ALT) and glutamic oxaloacetic transaminase (AST) activities in serum samples were measured, and haematoxylin and eosin (H&E) staining was performed to examine the liver tissue sections using routine methods. The animal experiments were approved by the Laboratory Animals Welfare Ethics Review Committee of Zhejiang University (ZJU20170522).

### 2.12. Statistical Analysis

GraphPad Prism software and R statistical software (version 4.1.1) were used for statistical analyses. All data are reported as the means ± SD (standard deviations) of at least three independent experiments. The significance of differences in the data was determined using the 2-tailed Student’s *t* test. *p*-values < 0.05 were considered significant.

## 3. Results

### 3.1. RRM2 Expression Was Positively Correlated with Malignant Proliferation in Patients with AML

**Figure 1:**
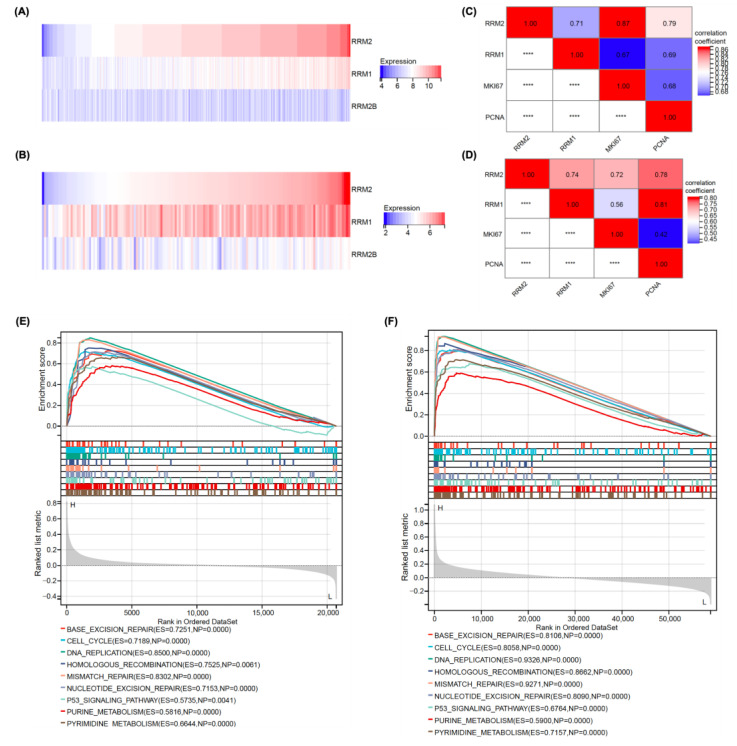
Analyses of RRM2 expression in samples from patients with AML from the GEO and TCGA databases. (**A**,**B**) Heatmaps showing the expression profiles of RRM1, RRM2, and RRM2B in patients with AML. (**C**,**D**) Correlation analyses between the expression of RRM2 and RRM1 with the cell proliferation biomarkers KI67 and PCNA in patients with AML. (**E**,**F**) KEGG enrichment plots constructed using GSEA for the RRM2 high expression group of patients with AML. Data in (**A**,**C**,**E**) were obtained from GSE147515 (N = 1534); data in (**B**,**D**,**F**) were obtained from TCGA (*n* = 151). **** is a mirror image of the chart and has no special meaning.

We downloaded the GSE147515 and TCGA datasets to investigate the role of RNR in patients with AML. The heatmaps depicted the expression profiles of the three RNR subunits, RRM1, RRM2 and RRM2B (Figure 1A,B). KI67 and PCNA are proliferation-related genes. Correlation analyses of the expression of these genes showed that RRM2 expression was significantly correlated not only with RRM1 expression, but also with KI67 and PCNA expression (Figure 1C,D). As a method to further understand the potential molecular mechanisms of RRM2 in AML, the AML samples were divided into RRM2 high and low expression groups, and GSEA was applied to identify the key biological pathways that were significantly correlated with the RRM2 high expression group. Among them, nine RNR function- and malignant phenotype-related pathways were highly enriched in the RRM2 high expression group, including the cell cycle, DNA replication, p53 signalling pathway, homologous recombination, nucleotide excision repair, base excision repair, mismatch repair, pyrimidine metabolism, and purine metabolism (Figure 1E,F). The results suggest a positive correlation between the RRM2 expression level and AML malignant proliferation, and RRM2 might be an important biomarker for risk stratification and therapeutic target for patients with AML.

### 3.2. MB Potently Inhibited RNR Enzymatic Activity by Interacting with the RRM2 Protein

**Figure 2:**
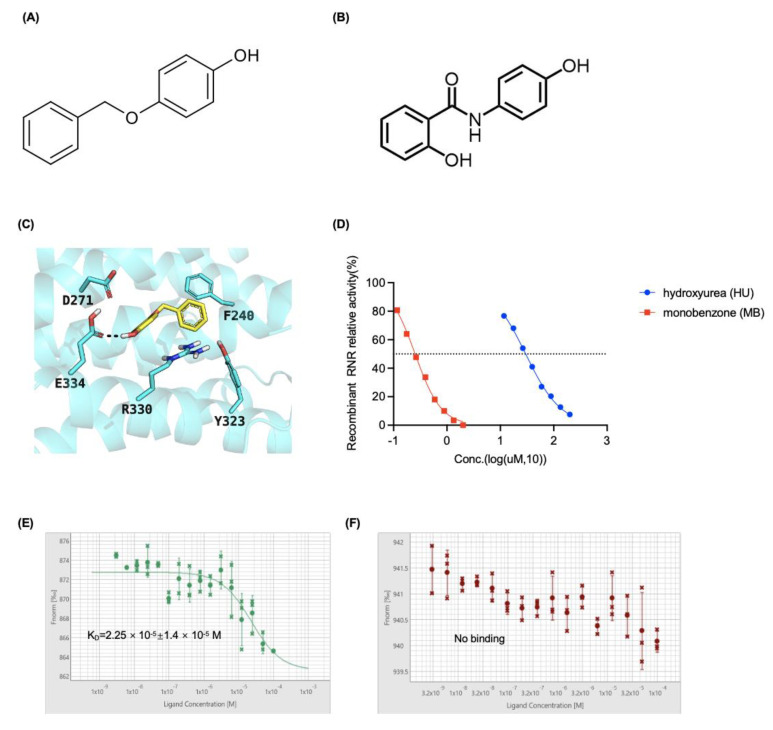
MB interacted with RRM2 and inhibited the activity of recombinant RNR in vitro. (**A**) Chemical structure of MB. (**B**) Chemical structure of osalmid. (**C**) The 3-dimensional interaction model between MB and RRM2 generated using molecular docking (Glide SP in Schrodinger/2020) showing the interaction between MB and key residues of RRM2 (PDB ID: 3OLJ). MB is represented in yellow sticks. RRM2 helices and residues are presented as a cyan cartoon and cyan sticks, respectively. Hydrogen bonds are represented as purple dashes. (**D**) In vitro recombinant RNR enzymatic assays. HU and MB dose-dependently inhibited the enzyme activity. The solvent DMSO was used as a negative control. (**E**,**F**) The binding between RRM2 protein and MB and the binding between RRM1 protein and MB were determined by MST assay. Fluorescence intensity and trend, and reproducibility of MST traces were shown. Data shown here are representative of three independent experiments, and the error is calculated as the standard deviation.

By virtual screening, we previously identified osalmid (Figure 2B) as a RRM2-targeting compound, which was 10-fold more active in inhibiting RR activity than hydroxyurea [43]. In this study, by performing similarity search, MB, having a 66.7% Tanimoto coefficient to osalmid was identified as a potential RRM2 inhibitor.

The binding mode of MB was predicted using molecular docking, and the results suggest that the ligand is a potential inhibitor of RRM2 (Figure 2A,C). By performing an enzymatic activity assay with recombinant RRM2 and RRM1 proteins, we showed that MB potently inhibited RNR activity in a dose-dependent manner, and the 50% inhibitory concentration (IC_50_) was 0.25 μM, which was approximately 100-fold lower than the IC_50_ of 29.82 μM for the RRM2-targeted drug HU (Figure 2D). Furthermore, MST analysis revealed an interaction between MB and RRM2 proteins with a KD value of 22.5 ± 14 μM (Figure 2E). Additionally, there is no interaction between MB and the large subunit RRM1 of RNR enzyme (Figure 2F). Based on these results, MB inhibits RNR activity by interacting with the RRM2 protein.

### 3.3. MB Effectively Inhibited Cell Growth and DNA Synthesis by Inhibiting RNR Enzymatic Activity in AML Cells

**Figure 3:**
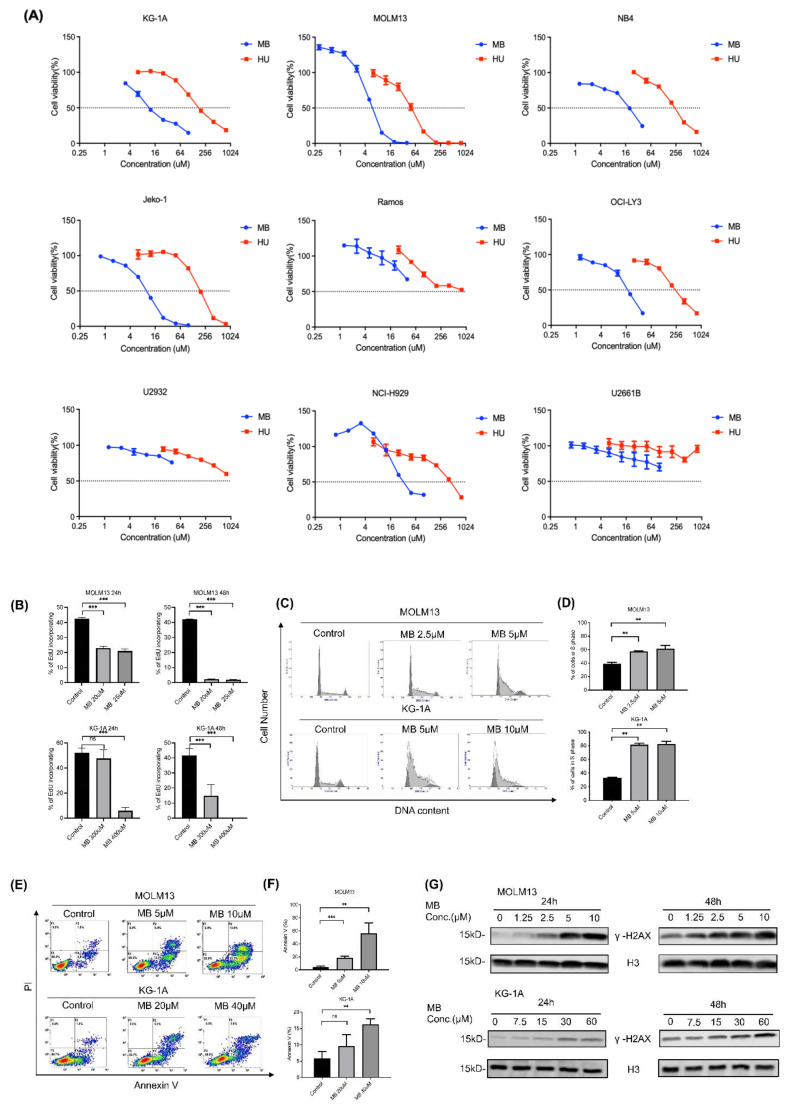

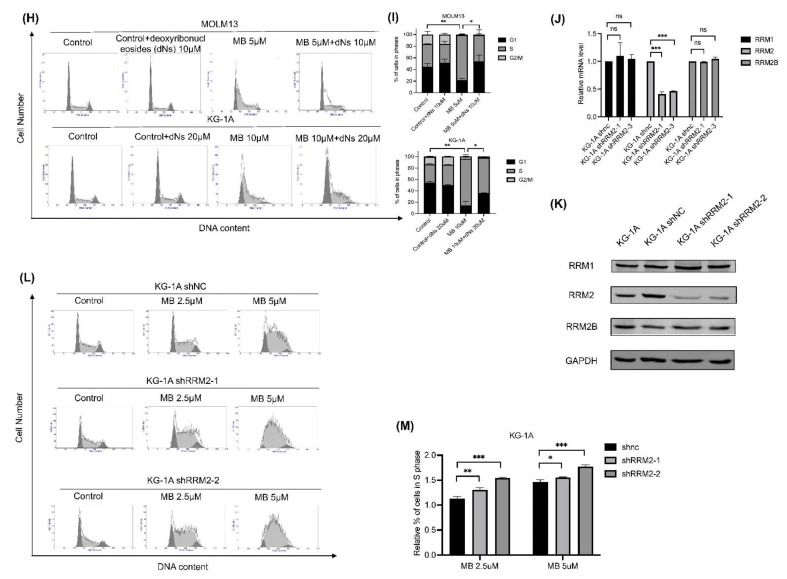
MB inhibited cell growth and DNA synthesis by inhibiting RNR enzymatic activity in AML cells. (**A**) MTT assays were performed on tumour cells treated with different doses of MB or HU for 72 h, and the solvent was used as negative control. (**B**) AML cells were treated with different doses of MB for 24 h or 48 h, and the EdU incorporation was examined with flow cytometry (FCM). (**C**,**D**) AML cells were treated with the indicated concentrations of MB for 24 h and then fixed with 70% ethanol, stained with PI, and subjected to an FCM analysis of the cell cycle distribution. The quantitative results of the number of cells in S phase are shown in a bar plot. (**E**,**F**) AML cells were treated with different doses of MB for 48 h and then analysed using FCM (PI & Annexin V method). Quantitative results of apoptotic cells are shown in bar plots. (**G**) AML cells were treated with MB for 24 h or 48 h. Whole-cell lysates were subjected to Western blotting with anti-γH2AX and anti-H3 antibodies. (**H**,**I**) AML cells were treated with MB alone or in combination with dNs for 24 h and then subjected to FCM analyses. The quantitative results of the numbers of cells in different phases of the cell cycle are shown in bar plots. (**J**) KG-1A cells were stably transfected with shRNAs targeting RRM2 (shRRM2-1 and shRRM2-2) or a negative control (shNC). The knockdown effects were measured with qRT-PCR. (**K**) The knockdown effects were measured with Western blotting. (**L**,**M**) Cells were treated with different doses of MB for 24 h and then subjected to FCM assays. The relative number of cells in the S phase is shown in a bar plot. All experiments described above were performed at least three independent times. Error bars represent the SD. * *p* < 0.05, ** *p* < 0.005, *** *p* < 0.0005 compared to the control group. Full Western blot images and western blotting gray value analysis can be found at Supplementary Figures S5 and S6, Tables S1 and S2.

Cell viability assays were performed to test the inhibitory effect of MB on tumour cells, including AML (KG-1A, MOLM13 and NB4), lymphoma (Jeko-1, Ramos, OCI-LY3 and U2932), and multiple myeloma (NCI-H929 and U2661B) cells. As shown in Figure 3A and Table 1, MB exerted a strong, dose-dependent inhibitory effect on the proliferation of treated AML cells, with IC_50_ values of approximately 6–18 μM. MOLM13 cells were the AML cell line most sensitive to MB, with an IC_50_ of approximately 6 μM, which was approximately one-tenth the IC_50_ of HU.

AML cells were treated with different concentrations of the compound to elucidate the mechanisms of action of MB. EdU incorporation analyses showed that MB dose-dependently inhibited DNA synthesis in MOLM13 and KG-1A cells (Figure 3B). Flow cytometry examinations showed that the compound induced cell cycle is arrested at the S phase (Figure 3C,D) and apoptosis (Figure 3E,F) in AML cells. Furthermore, Western blotting for r-H2Ax showed that MB blocked DNA damage repair in the treated cells (Figure 3G). Importantly, the addition of exogenous dNs (including thymidine, deoxyadenosine, deoxyguanosine, and deoxycytidine) as dNTP precursors significantly reversed the cell cycle arrest in S phase caused by MB treatment at a concentration ratio of 2:1 in the AML cells (Figure 3H,I), suggesting that RNR is the active target of the compound in the treated cells, resulting in the inhibition of DNA synthesis and cell proliferation. In addition, knockdown of RRM2 alone with specific shRNAs (Figure 3J,K) enhanced the RNR inhibitory effects of MB, as shown by the S phase arrest analyses in AML cells (Figure 3L,M), supporting the hypothesis that targeting intracellular RRM2 might inhibit AML malignant behaviours.

### 3.4. MB Overcame the Drug Resistance to Ara-C, DOX and HU in AML Cells

**Figure 4:**
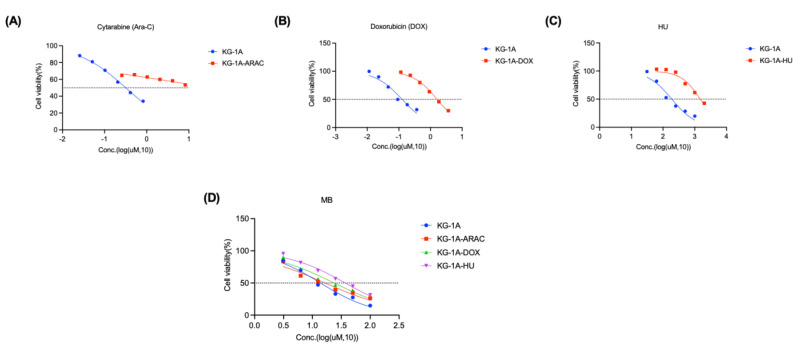
MB overcame drug resistance in AML cells. (**A**–**C**) KG-1A cells were treated with different doses of the compounds for 72 h and then cell viability was measured using MTT assays. (**D**) The antiproliferative effects of MB on the parental and resistant KG-1A cells were determined by performing MTT assays. Error bars represent SD. The resistant ratio = IC_50_ for resistant cells/IC_50_ for parent cells.

Drug resistance is a major problem in clinical AML treatment. We constructed Ara-C-, DOX-, and HU-resistant KG-1A cell lines (KG-1A-ARAC, KG-1A-DOX, and KG-1A-HU). The IC50s of these drugs for AML cells were increased at least 7-fold (Figure 4A–C and Table 2). In comparison, MB inhibited the growth of these drug-resistant cells and their parental cells with a similar potency in a dose-dependent manner (Figure 4D and Table 3).

### 3.5. Combination of MB with the BCL-2 Inhibitor ABT-737 Resulted in Synergistic Inhibitory Effects on AML Cells

**Figure 5:**
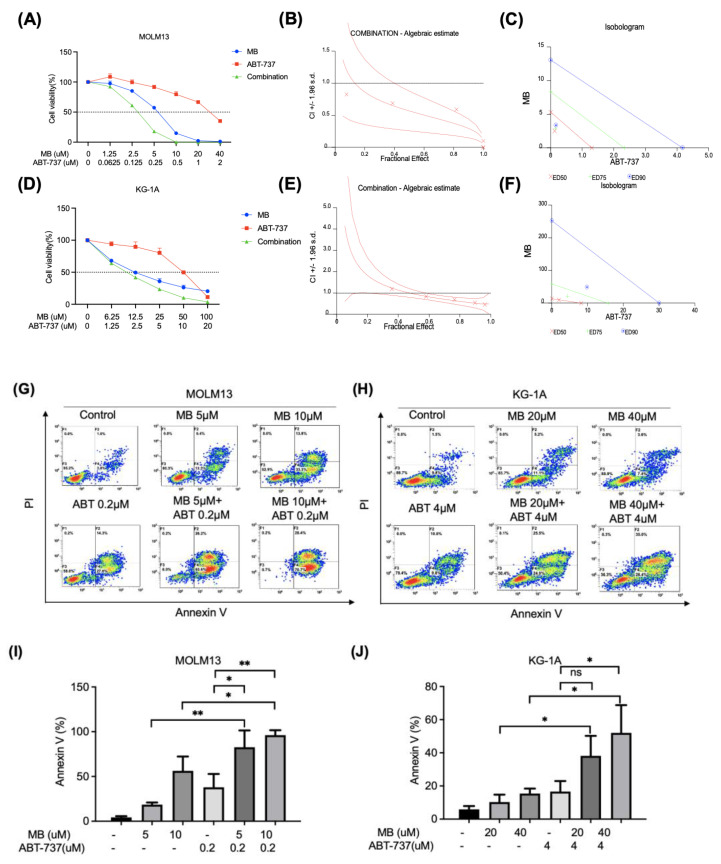
Treatment with a combination of MB and the BCL-2 inhibitor ABT-737 resulted in synergistic inhibition of AML cell growth. (**A**,**D**) AML cells were treated with different doses of MB or ABT-737 or their combination for 72 h and analysed with MTT assays. (**B**,**E**) The effects of the combination treatment were assessed using the CalcuSyn program. For the Fractional Effect-Combination Index plots, the Y-axis represents the combination index (CI), and the X-axis represents the magnitude of effect (maximum 1.0). The middle line represents a plot of the data and the other two lines show the confidence intervals. CI <1 indicates a synergistic effect, CI = 1.0 indicates an additive effect, and CI >1 indicates an antagonistic effect. (**C**,**F**) For the isobologram plots, the three diagonal straight lines connecting the ED50, ED75, and ED90 of MB and ABT-737 represent the theoretical lines of additivity for a continuum of different fixed dose ratios. The three single points representing the combinations at ED50, ED75, and ED90 under their respective diagonal straight lines denote synergism. (**G**–**J**) AML cells were treated with different doses of MB for 48 h and then analysed for apoptosis using FCM (PI and Annexin V method). Results from the quantitative analysis of the percent of apoptotic cells (Annexin V+) are shown in bar blots. Error bars represent SD. * *p* < 0.05, ** *p* < 0.005 compared to the single agent group.

The anti-apoptotic Bcl-2 gene was expressed at high levels in patients with AML compared to normal people in the GSE147515 and TCGA databases (Figure S3). The combination of antiproliferative RNR inhibitors with proapoptotic agents may strengthen the efficacy against AML. We tested this possibility by mixing MB with the Bcl-2 inhibitor ABT-737 (at a 20:1 and 5:1 ratio according to their respective IC_50_s for MOLM13 and KG-1A cells, respectively) to treat AML cells. The cell viability assays showed that MB and ABT-737 synergistically inhibited the growth of AML cells (Figure 5A–F and Table 4). The IC50s of MB and ABT-737 decreased from 5.40 μM to 2.52 μM and from 1.41 μM to 0.13 μM, respectively, in MOLM13 cells, as well as from 13.66 μM to 9.69 μM and from 9.37 μM to 2.13 μM, respectively, in KG-1A cells. The combination treatments also caused more significant cell apoptosis than either compound alone in AML cells (Figure 5G–J). Thus, the combination of MB and ABT-737 exerted a synergistic inhibitory effect on AML cells.

### 3.6. MB Effectively Inhibited AML Cell Xenograft Growth in Nude Mice with Relatively Low Toxicity

**Figure 6:**
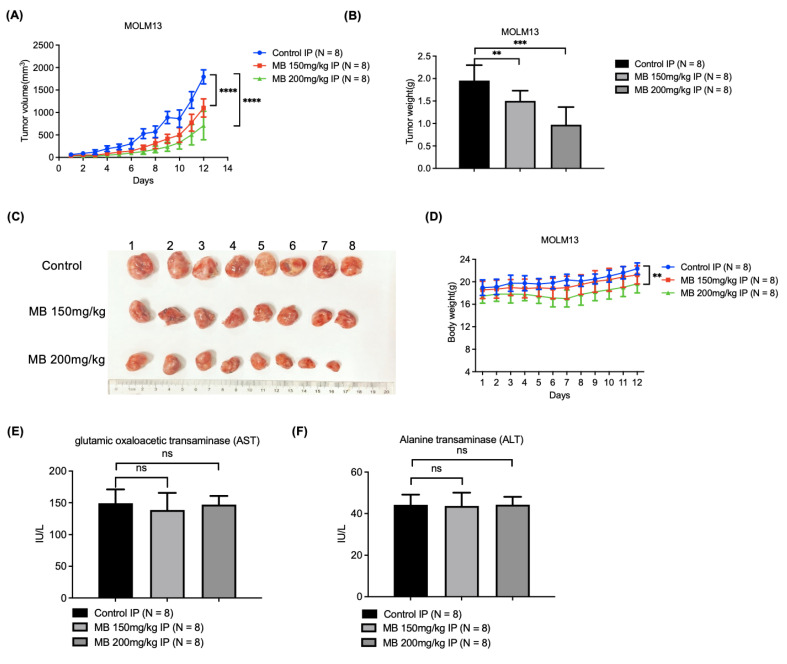
MB effectively inhibited the growth of AML cell xenografts in nude mice with relatively low toxicity. (**A**,**B**) The sizes and weights of tumour xenografts in nude mice from different treatment groups (N = 8). (**C**) Images of the tumour xenografts. (**D**) The body weights of mice in different groups. (**E**,**F**) Serum AST and ALT levels in each mouse from different groups. IP: Intraperitoneal injection. Error bars represent SD. ** *p* < 0.005, *** *p* < 0.0005 compared to the control group.

We evaluated the anti-AML activity of MB in vivo by constructing a subcutaneous xenograft of MOLM13 cells in mice. The mice were treated with two doses of MB by intraperitoneal injection once a day for two weeks. The measured tumour volumes and weights showed that MB significantly reduced AML cell growth in nude mice compared with the solvent control (Figure 6A–C). In contrast, the body weights, serum alanine transaminase (ALT) and glutamic-pyruvic transaminase (AST) levels, and HE staining of heart, liver, spleen, lung and kidney tissues were not significantly different between the MB-treated mice and the solvent control group (Figure 6D–F and Figure S4), except for a small body weight loss in the group treated with the higher dose (Figure 6D). Although the weight of the mice in the high-concentration MB treatment group grew a little slower than that in the control group, it still showed a continuous and steady upward trend. At the same time, we observed no significant difference in general activity and diet conditions between the control and treated mice, indicating that the toxic effects of MB on the mice were within acceptable range. Based on these results, MB effectively inhibited AML cell growth in vivo with relatively low toxicity.

## 4. Discussion

AML is a highly proliferative haematological malignancy and still lacks effective drugs with low toxicity and high specificity; additionally, drug resistance frequently leads to treatment failure and recurrence of the disease. RNR is a rate-limiting enzyme required for DNA synthesis that regulates the supply of dNTPs, thereby controlling cell proliferation and playing an important role in cancer development. In the present study, the expression of RNR subunits, especially RRM2, was strongly correlated with malignant proliferation in a large cohort of patients with AML in the GEO and TCGA databases, suggesting a rationale for inhibiting RNR to treat AML. Based on the 3D structure of the RRM2 protein, computer-assisted molecular docking predicted MB, an FDA-approved external skin medication for patients with vitiligo, as a potential inhibitor of RNR. Recombinant RNR activity and MST assays showed that MB potently inhibited the enzyme by interacting with the RRM2 protein in vitro. The reduced EdU incorporation, upregulated r-H2Ax level, increased cell cycle arrest in S phase and reversibility by dNTP precursors, the dose-dependent cell viability inhibition and apoptosis induction together indicated that the compound inhibited DNA synthesis and thereby cell proliferation by inhibiting RNR activity in treated AML cells. Furthermore, the compound significantly inhibited the growth of AML cell xenografts in nude mice with relatively low toxicity in vivo. Thus, this study reveals that MB is a novel potent anti-AML agent that inhibits RNR.

The skin-depigmenting effect of MB is correlated with the inhibition of tyrosinase, the rate-limiting enzyme in melanin synthesis, in both melanocytes and melanoma cells [30,31]. The metabolites of MB have been proven to exert toxic effects on melanocytes and increase melanocyte and melanoma cell immunogenicity [32,33,34,35]. In a phase 2 clinical trial, topical treatment with MB and imiquimod (Toll-like receptor agonist) induced local and systemic antimelanoma immunity and local regression of inoperable cutaneous metastases in patients with stage III-IV melanoma, and the treatment was well tolerated [36]. The study by Peizhi Ma et al. showed that MB can inhibit KDM1A activity and cancer progression in two LSD1 overexpressed gastric cell lines in vitro [45]. In our study, we demonstrated that MB potently inhibited RNR enzyme activity by targeting RRM2 and thereby suppressed AML cell growth in vitro and in a mouse xenograft experiment. As shown by DepMap, our previous analysis also showed that the expression of RRM2 is abnormally highly increased in multiple cancers [46], supporting that it is essential for cancer development. Although MB is a pleiotropic inhibitor, RRM2 is one of the most important targets for the compound’s activity. In the present study, we examined the activity and expression of tyrosinase in leukaemia cells [44]. As shown in Supplementary Figures S1 and S2 (Full Western blot images and western blotting gray value analysis can be found at Supplementary Figure S7 and Table S3), tyrosinase activity and protein levels were extremely low in the haematological tumour cells compared with those in the mouse melanoma cell line B16. On the other hand, MB inhibited RNR activity, AML cell growth, and AML xenografts in immunodeficient nude mice. Thus, the anti-AML effect of MB is not dependent on tyrosinase expression or the immune response, but its mechanism of action is to target RNR in AML cells.

Ara-C represents a prototype of the nucleoside analogue class of antineoplastic agents and remains one of the most effective drugs used to treat AML and other haematopoietic malignancies. Ara-C is a substrate for deoxycytidine kinase (dCK) and is metabolized into ara-CDP and ara-CTP in cells. Ara-CDP inhibits RNR activity as a substrate analogue of RRM1, while ara-CTP inhibits DNA synthesis after it is incorporated into DNA by DNA polymerase. However, neoplastic cells frequently become resistant to Ara-C through a wide variety of mechanisms, such as decreased activity of dCK and enhanced deamination or dephosphorylation [47]. DOX, similar to daunorubicin, inhibits RNA and DNA synthesis and is often combined with Ara-C for the treatment of AML. DOX exhibits cross drug resistance with daunorubicin. In this study, MB showed approximately equal efficacy in overcoming Ara-C and DOX resistance in AML cells, possibly because they belong to different categories of compounds and possess different targeting mechanisms.

HU is a typical RNR inhibitor that targets RRM2 and has long been used for cancer therapy in the clinic, including chronic myelogenous leukaemia, AML, and other haematological malignancies. However, its effectiveness is limited by its low specificity, small size, short half-life, and development of resistance [23]. Due to the small molecular weight of HU (MW 76.0547), its binding specificity with RRM2 protein is poor. There is currently no evidence that HU binds to the E. coli R2 or human RRM2 proteins [48,49,50]. Previous studies have shown that HU inactivates RNR by reducing the tyrosyl radical and diiron centre of RRM2 [22], or by interfering with the interface of RNR small and large subunits [51,52]. From the docking diagram of our experimental results, it can be seen that RRM2 E334 and MB are bound by a hydrogen bond (represented by black dashed lines), and the D271 forms polar interaction with MB and the F240 provides Π-Π conjugation. Other key amino acid residues involved in non-bonding interactions are represented by rod-like structures, and the rest of the protein is represented by cartoons. The D271 and E334 are located in the second half of helix 9 and 12 of RRM2, respectively. The predicted binding site of MB is adjacent to the active centre of RRM2, i.e., the ferritin-like diiron-binding domain of RRM2, suggesting that the binding of MB may interfere with electron transport between RRM2 and RRM1 and thereby inhibit RNR activity. In this study, MB showed a much higher RNR inhibitory and antiproliferative potency than HU and overcame the resistance of AML cells to HU, suggesting that MB is a new category of RNR inhibitory compounds with potent activity against AML.

The antiapoptotic BCL-2 family members inhibit apoptosis mainly by binding through the BH3 domain to and thereby suppressing the activity of the proapoptotic proteins Bax and Bak. ABT-737 is a small-molecule BH3 mimetic that induces apoptosis by inhibiting the interaction between some of the antiapoptotic and proapoptotic proteins and has been reported to possess strong antitumour activities against AML and other tumour cells [53]. In this study, we showed that BCL-2 expression was substantially upregulated in samples from patients with AML in a public database. Treatment with the combination of MB and ABT-737 exerted a synergistic inhibitory effect on AML cells. Therefore, the simultaneous targeting of the pre-proliferative protein RNR and the antiapoptotic protein BCL-2 may rationally enhance therapeutic efficacy in the clinical treatment of patients with AML.

## 5. Conclusions

We show a positive correlation between increased RNR expression and proliferative malignancy in patients with AML in this study, and MB potently inhibits RNR activity by targeting the RRM2 protein and thereby blocks DNA synthesis and the malignant proliferation of AML cells in vitro and in vivo. MB overcomes drug resistance to the common AML drugs Ara-C and DOX and to the representative RRM2 inhibitor HU. Moreover, the antiproliferative RNR inhibitor MB and the proapoptotic Bcl-2 inhibitor ABT-737 generate a synergistic therapeutic effect on AML cells, suggesting that rational combination treatment may also exert synergistic effects on other malignancies. Thus, we propose that MB represents a new category of RNR inhibitory agents with the potential for the development of novel anti-AML drugs in the future.

## Figures and Tables

**Table 1:** Statistics of MTT IC_50_ values.

IC_50_ (μM)	MB	HU
KG-1A	13.9 ± 1.64	201.05 ± 16.75
MOLM13	6.07 ± 1.55	48.85 ± 3.38
NB4	18.04 ± 3.36	242.6 ± 14.2
Jeko-1	9.6 ± 0.44	192.85 ± 11.45
Ramos	62.95 ± 29.02	715.65 ± 327.35
U2932	302.3 ± 156	1517 ± 309
OCI-LY3	16.96 ± 1.57	249.85 ± 16.55
NCI-H929	42.86 ± 14.68	422.85 ± 67.95
U2661B	>100.00	>800.00

**Table 2:** Statistics of MTT IC_50_ values.

IC_50_ (μM)	Parental	Resistant	Resistant Ratio
Ara-C	0.32 ± 0.03	33.04 ± 14.36	103.25
DOX	0.12 ± 0.02	1.53 ± 0.1	12.64
HU	189.3 ± 34.3	1498 ± 179	7.90

**Table 3:** Statistics of MTT IC_50_ values.

IC_50_ (μM)	MB	Resistant Ratio
KG-1A	13.9 ± 1.64	1
KG-1A-ARAC	16.88 ± 3.08	1.20
KG-1A-DOX	24.25 ± 3.82	1.74
KG-1A-HU	37.04 ± 4.38	2.68

**Table 4:** Synergistic inhibitory effects of MB and ABT-737 on AML cells.

Compound		IC_50_ of Cell Viability Inhibition(μM)	
		MOLM13	KG-1A
Single	MB	5.40 ± 0.22	13.66 ± 1.71
	ABT-737	1.41 ± 0.17	9.37 ± 0.97
Combination	MB	2.52	9.69
	ABT-737	0.13	2.13

## Data Availability

The data acquired from the public dataset are available at: https://portal.gdc.cancer.gov and https://xenabrowser.net/datapages/.

## Supplementary Materials

The following supporting information can be downloaded at: https://www.mdpi.com/article/10.3390/cancers14194710/s1, Figure S1: Tyrosinase activity assays; Figure S2: Western blot showing tyrosinase protein levels in the indicated cells; Figure S3: BCL-2 expression in samples from normal persons (N = 198) and patients with AML (N = 1534) in the GSE147515 dataset; Figure S4: HE staining of mouse hearts, livers, spleens, lungs and kidneys from different groups; Figure S5: original blots of Figure 3G; Figure S6: original blots of Figure 3K; Figure S7: original blots of Figure S2; Table S1. Western blotting gray value analysis of Figure 3G; Table S2. Western blotting gray value analysis of Figure 3K; Table S3. Western blotting gray value analysis of Figure S2.
