# Supplementary material for: Identification of Monobenzone as a Novel Potential Anti-Acute Myeloid Leukaemia Agent That Inhibits RNR and Suppresses Tumour Growth in Mouse Xenograft Model
Source: Cancers (Basel). 2022 Sep 27;14(19):4710. doi: 10.3390/cancers14194710 (PMC9564123; doi:10.3390/cancers14194710)
Supplement: Supplementary file 1 [file cancers-14-04710-s001.zip › cancers-1883718-supplementary(1).pdf]

Supplementary Materials

Identification of monobenzone as a novel potential anti-acute myeloid leukaemia agent that inhibits RR and suppresses tumor growth in mouse xenograft model

Jingwen Dong, Tingting Zhong, Zhijian Xu, Haiyi Chen, Xianjun Wang, Lili Yang, Zhiyuan Lou, Yuanling Xu, Tingjun Hou, Rongzhen Xu, Weiliang Zhu and Jimin Shao

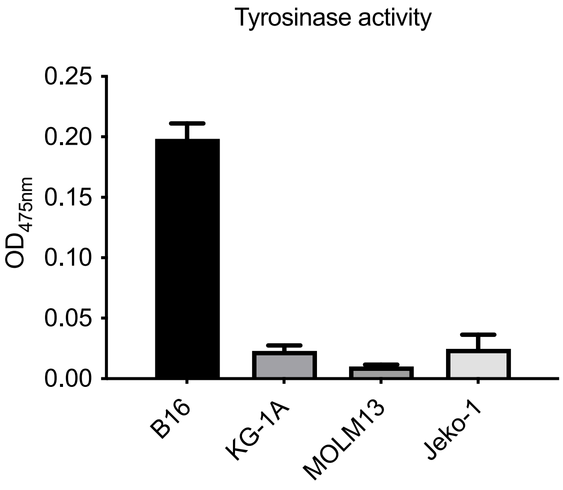

Figure S1. Tyrosinase activity assays. After the indicated cells were treated with L-dopa, the absorbance was measured at 475 nm.

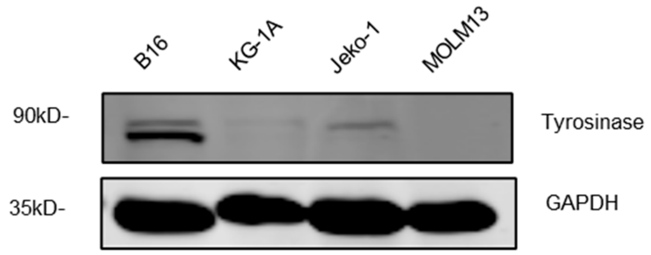

Figure S2. Western blot showing tyrosinase protein levels in the indicated cells. Relative protein level of tyrosinase and GAPDH were shown.

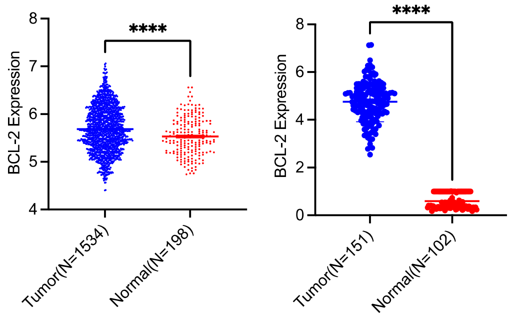

Figure S3. BCL-2 expression in samples from normal persons (N = 198) and patients with AML (N = 1534) in the GSE147515 dataset. BCL-2 expression in samples from normal persons (bone marrow samples) (N = 102) in the GTEx dataset and patients with AML (N = 151) in TCGA dataset. Error bars represent SD. *p < 0.05 represents the difference between the tumor group and the normal group.

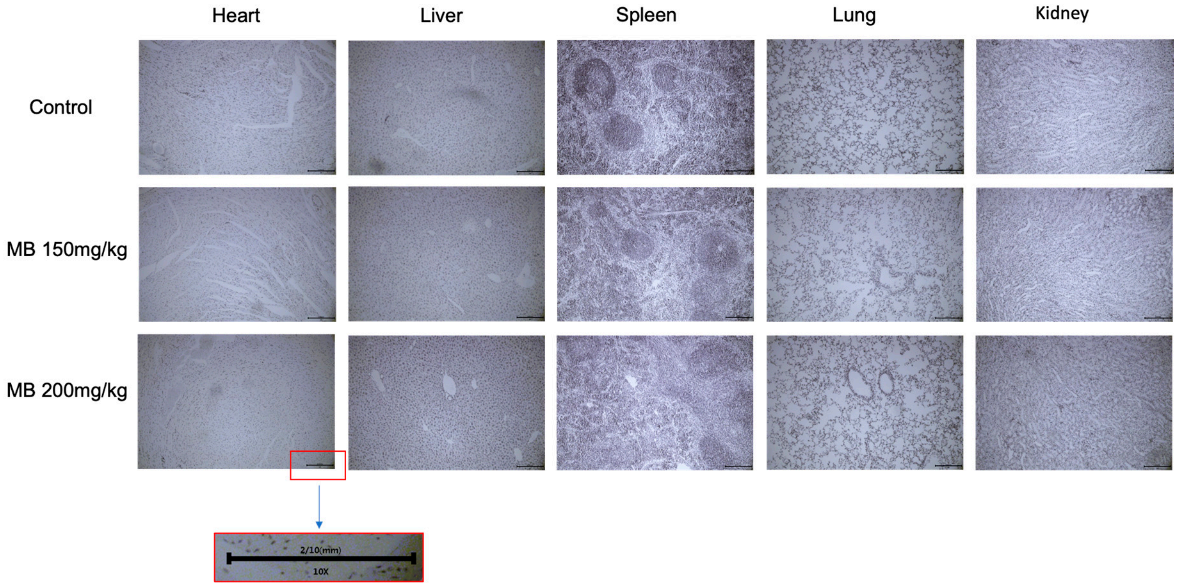

Figure S4. HE staining of mouse hearts, livers, spleens, lungs and kidneys from different groups.

Figure 3G

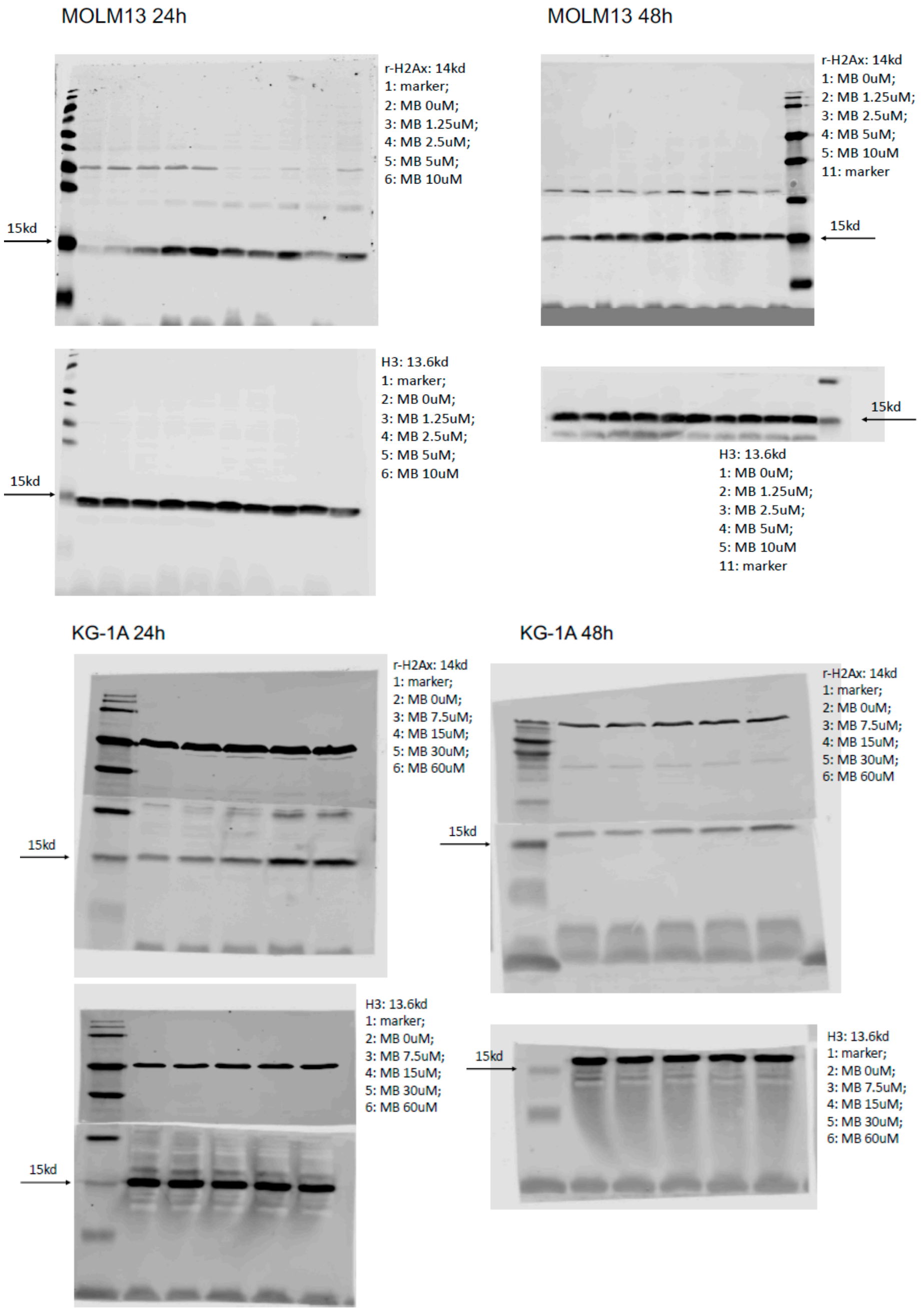

Table 1. Western blotting gray value analysis of figure 3G

	MOLM13 24h					MOLM13 48h				
	Control	MB 1.25uM	MB 2.5uM	MB 5uM	MB 10uM	Control	MB 1.25uM	MB 2.5uM	MB 5uM	MB 10uM
r-H2Ax	162	1278	5759	15810	17793	6524	10705	12051	13491	17165
H3	15923	25954	18960	24630	14956	22528	10616	15083	14503	9810
	KG-1A 24h					KG-1A 48h				
	Control	MB 7.5uM	MB 15uM	MB 30uM	MB 60uM	Control	MB 7.5uM	MB 15uM	MB 30uM	MB 60uM
r-H2Ax	9667	13158	16135	17619	25023	10137	11756	13949	25840	19350
H3	24258	21458	24016	24208	28439	15289	22103	20835	23657	24404

Figure S5. original blots of Figure 3G.

Figure 3K

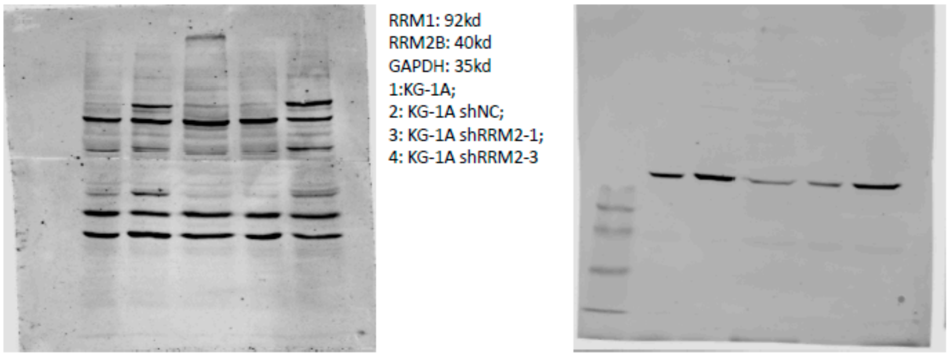

Table 2. Western blotting gray value analysis of figure 3K

	KG-1A	KG-1A shNC	KG-1A shRRM2-1	KG-1A shRRM2-3
RRM1	54901	67140	90352	65045
RRM2	38980	59762	21152	15359
RRM2B	55696	45854	64969	43021
GAPDH	61835	77188	78652	73806

Figure S6. original blots of Figure 3K.

Figure S2

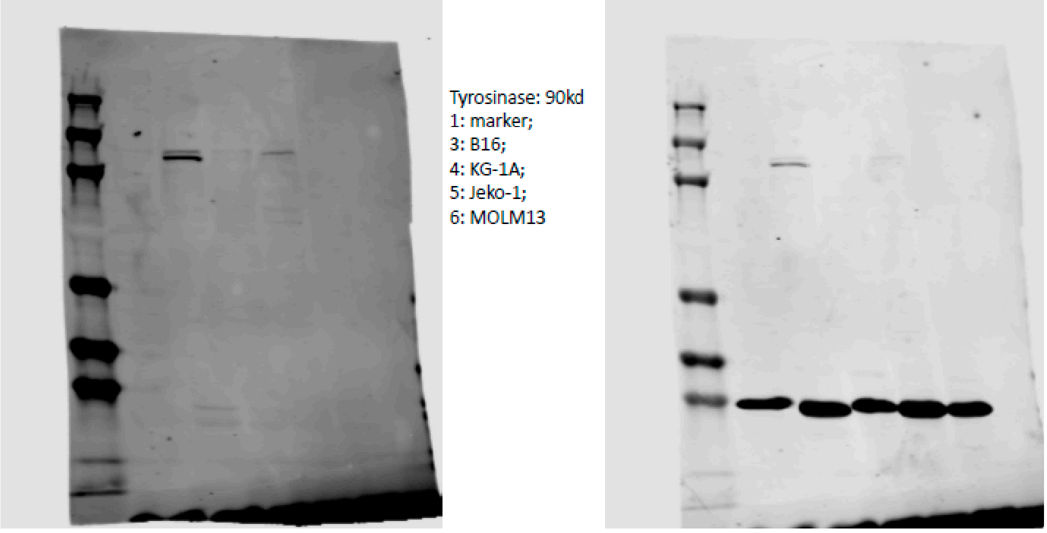

Table 3. Western blotting gray value analysis of figure S2

	B16	KG-1A	Jeko-1	MOLM13
Tyrosinase	50548	4656	12977	2112
GAPDH	54993	28993	42095	41541

Figure S7. original blots of Figure S2.
